# The efficacy of immune checkpoint inhibitors in advanced *EGFR*-Mutated non-small cell lung cancer after resistance to EGFR-TKIs: Real-World evidence from a multicenter retrospective study

**DOI:** 10.3389/fimmu.2022.975246

**Published:** 2022-09-09

**Authors:** Jia Hu, Di Huang, Yanrong Wang, Donghui Li, Xuejiao Yang, Yan Fu, Nan Du, Yan Zhao, Xiaosong Li, Junxun Ma, Yi Hu

**Affiliations:** ^1^ Department of Oncology, The Fifth Medical Center of PLA General Hospital, Beijing, China; ^2^ Department of Oncology, The Seventh Medical Center of PLA General Hospital, Beijing, China; ^3^ Department of Oncology, The Fourth Medical Center of PLA General Hospital, Beijing, China; ^4^ Department of Oncology, The First Medical Center of PLA General Hospital, Beijing, China

**Keywords:** immune checkpoint inhibitor, epidermal growth factor receptor (*EGFR*) mutation, epidermal growth factor receptor tyrosine kinase inhibitor (EGFR) TKI, non-small cell lung cancer, combination therapy

## Abstract

**Background:**

The efficacy of immune checkpoint inhibitors (ICIs) in pretreated *EGFR*-mutated non-small cell lung cancer (NSCLC) patients is controversial. We conducted this multicenter retrospective study to examine the efficacy of ICIs in a real world setting.

**Patients and methods:**

We collected 116 consecutive NSCLC patients with sensitive *EGFR* mutations who received ICIs alone or in combination after failure to respond to EGFR tyrosine kinase inhibitors (EGFR-TKIs), and 99 patients were included for final analysis. The impacts of ICIs on the patients’ objective response rate (ORR), disease control rate (DCR), progression-free survival (PFS), and overall survival (OS) were assessed. The relationships between outcomes and clinical characteristics were analyzed.

**Results:**

The ORR in patients with target lesions was 31.25% (95% CI: 22.18-41.52), and the DCR in all patients was 65.66% (95% CI: 55.44-74.91). The overall median PFS was 5.0 months (95% CI: 3.0-6.6), and the median OS was 15.9 months (95% CI: 10.8-23.8). The outcomes were better in patients receiving combination therapy with ECOG scores of 0-1 and no more than 2 lines of prior therapy, with a median PFS of 7.4 months (95% CI: 3.0-13.3) and a median OS of 29.0 months (95% CI: 11.7-NE). Primary *EGFR* mutation type and treatment mode were found to have a notable impact on clinical outcomes. Both median PFS and OS in patients with *EGFR* L858R mutation were significantly shorter than those in patients with *EGFR* exon 19 deletion (19del) (PFS: 2.5 versus 6.7 months, HR: 1.80, log-rank *P*=0.011; OS: 9.8 versus 26.9 months, HR: 2.48, log-rank *P*=0.002). Patients receiving combination therapy had notably longer median PFS and OS than those receiving monotherapy (PFS: 5.2 versus 3.0 months, HR: 0.54, log-rank *P*=0.020; OS: 19.0 versus 7.4 months, HR: 0.46, log-rank *P*=0.009).

**Conclusions:**

Our study suggests that ICI-based combination therapy is a potential strategy for *EGFR*-mutated NSCLC patients after EGFR-TKI failure. The efficacy may differ according to *EGFR* subtypes.

## 1 Introduction

The discovery of epidermal growth factor receptor (*EGFR*) mutations and the advent of EGFR-tyrosine kinase inhibitors (TKIs) have dramatically shifted the therapeutic landscape of non-small cell lung cancer (NSCLC) from traditional chemotherapy to molecular targeted therapy. Characterized by low toxicity and high efficiency, EGFR-TKIs have become the standard of care as first-line treatment for patients with sensitive mutations (1, 2). However, resistance to targeted therapy is inevitable (3, 4). Although the development of third-generation EGFR-TKIs has overcome approximately 60% of acquired resistance due to the *EGFR* T790M mutation (5, 6), challenges still exist in treatments after failure of third-generation TKIs or first- and second-generation TKIs without T790M. Progression-free survival (PFS) with traditional chemotherapy in the subsequent lines of treatment is a disappointing length of 4.4-5.4 months (7, 8). New strategies are urgently needed to improve the prognosis of patients who are resistant to EGFR-TKI therapy.

In recent years, tremendous advances in immune checkpoint inhibitors (ICIs) have significantly improved the overall survival of advanced NSCLC patients without driver mutations, while the efficacy of programmed death 1 (PD-1) axis inhibition in *EGFR*-positive patients is still controversial. As programmed cell death-ligand 1 (PD-L1) levels have been reported to be significantly higher in *EGFR*-mutated NSCLC cell lines, especially in EGFR TKI-resistant cells (9–11), and EGFR-TKI treatment is considered to be associated with an increase in TMB and PD-1 expression (12), PD-1/PD-L1 blockade is considered a promising approach in NSCLC with *EGFR* mutations, especially in patients with acquired resistance to EGFR-TKIs. However, data from clinical trials have not shown substantial survival benefits of single-agent ICIs in pretreated *EGFR*-mutated NSCLC (13–19). Furthermore, the high rate of interstitial pneumonitis discontinued the attempt to combine of EGFR-TKI and PD-1 blockade (20).

With the further understanding of the synergistic mechanism of chemotherapy and immunotherapy, as well as the release of data from the clinical trial Impower150, more studies are focusing on the efficacy of immunotherapy-based combination treatment in *EGFR*-mutated NSCLC after EGFR-TKI resistance. In phase II trials, toripalimab and tislelizumab were reported to have objective response rates (ORRs) of 50.0% and 59.4% and disease control rates (DCRs) of 87.5% and 90.6%, respectively, in combination with chemotherapy as second-line treatment in pretreated *EGFR*-mutated advanced NSCLC (21, 22). Several phase III trials are ongoing. However, some limitations exist. First, their sample sizes were small. Second, it was not clear which patients would benefit most from this immunotherapy-based combination treatment. Finally, comparisons between single-agent ICIs and combination therapy were absent. To overcome these problems, we conducted this study to show the efficacy of ICIs in pretreated advanced *EGFR*-mutated NSCLC in a real world setting.

## 2 Methods

### 2.1 Study population

This was a multicenter, retrospective, cohort analysis of consecutive advanced NSCLC patients with sensitive *EGFR* mutations who received ICIs after resistance to EGFR-TKIs. Sensitive *EGFR* mutations included *EGFR* exon 19 deletion (19del), L858R, exon 19 insertion, L861Q, G719X and S768I. Data were collected from patients treated in the First, Fourth and Fifth Medical Center of PLA General Hospital between 2018 and 2021. Patients who were histologically confirmed to have advanced NSCLC with a sensitive *EGFR* mutation identified by polymerase chain reaction (PCR) testing or next generation sequencing (NGS) were eligible for inclusion. They must have received at least one line of EGFR-TKI therapy before receiving ICIs and experienced either disease progression or toxicity requiring a change in systemic therapy. Patients who received ICIs with biopsy-proven small cell transformation after progression or without complete clinical information were excluded. The Ethics Committee of PLA General Hospital approved this study (S2018-092-01).

### 2.2 Data collection

Data were collected from the electronic medical record system in each medical center. Demographic, clinicopathological and treatment data were collected with uniform database templates to ensure consistent data collection. For ICIs, including anti-PD-1/PD-L1 antibody, information on the specific drug, treatment time, reason for discontinuation, and grade 3 and above treatment-related adverse events (TRAEs) or any grade immune-related adverse events (irAEs) were collected. According to the response evaluation criteria in solid tumors (RECIST 1.1), radiographic responses were classified into four categories for patients with target lesions: complete response (CR), partial response (PR), stable disease (SD) or progressive disease (PD). And for patients had only non-target lesions, the assessments were classified into CR, NonCR/NonPD or PD.

### 2.3 Study outcomes

The main objective of this study was to evaluate the efficacy of ICIs in NSCLC patients with sensitive *EGFR* mutations after failure to respond to EGFR-TKI therapy. The impacts of ICIs on ORR, DCR, PFS, and overall survival (OS) were assessed. ORR was defined as the proportion of patients with target lesions who achieved CR or PR as the best radiographic response. The DCR included the proportion of all the patients without PD. PFS was calculated from the initiation of ICI-based therapy until disease progression, death or the last follow-up, while OS was calculated until death or the last follow-up. Patients data were limited to the date of last dose if they discontinued treatment because of nonprogressive disease causes without further disease assessment.

### 2.4 Statistical analysis

Data were analyzed using STATA statistical software version 17.0. Clinical characteristics and safety data were summarized by descriptive statistical analysis. The differences in tumor response (objective response and disease control) in different subgroups were assessed using the chi-square test or Fisher’s exact test. Analyses of PFS and OS were performed using the Kaplan-Meier method. Univariate analysis and multivariate analysis were conducted to assess predictive factors associated with PFS or OS *via* Cox proportional hazard modeling. Variables that were selected for multivariate analysis included age, gender, Eastern Cooperative Oncology Group (ECOG) score, smoking history, primary *EGFR* mutation type, prior treatment of third-generation EGFR-TKI, status of brain metastasis, status of liver metastasis, status of bone metastasis, prior lines of therapy and treatment mode, and a backward stepwise regression procedure was applied. The hazard ratio (HR) was estimated to compare survival according to the factor of interest and significance was determined by the log-rank test. *P* values <0.05 were considered to be statistically significant.

## 3 Results

### 3.1 Patient characteristics and treatment

One hundred and sixteen NSCLC patients harboring sensitive *EGFR* mutations, who were treated with ICIs after EGFR TKI progression were identified from three institutions. Five patients were excluded for biopsy-proven small cell transformation after progression, and 12 were excluded for insufficient clinical information. Finally, a total of 99 patients were enrolled in this study for further analysis (**Supplementary Figure S1**). The median age at initiation of treatment with ICIs was 59 years (range from 34 to 92) and 44 were male. Primary *EGFR* mutations included 50 cases of 19del, 42 of L858R mutation, 3 of L861Q, 3 of G719X and 1 of S768I. Ninety-three patients received anti-PD-1 antibody and 6 received anti-PD-L1 antibody. Twenty patients (20.20%) received monotherapy while 79 (79.80%) received combination therapy. Combination therapy included immunotherapy-chemotherapy combination treatment (I+C: n=27; 27.27%), immunotherapy-antiangiogenic combination treatment (I+A: n=19; 19.19%), immunotherapy-antiangiogenic-chemotherapy combination treatment (I+A+C: n=28; 28.28%), PD-1 inhibitor combined with cytotoxic T-cell lymphocyte-4 (CTLA-4) inhibitor (n=1; 1.01%), and PD-1 inhibitor combined with EGFR TKI (n=4; 4.04%). The detailed characteristics of these patients are listed in **Table 1**.

**Table 1:** Characteristics of patients with sensitive *EGFR* mutations receiving ICIs.

Characteristic	All patients (N = 99)
Median age (range), y	59(34-92)
Gender, n (%)	
Male	44 (44.44)
Female	55 (55.56)
ECOG score, n (%)	
0-1	70 (70.71)
≥2	29 (29.29)
Smoking history, n (%)	
Current or former	22 (22.22)
Never	77 (77.78)
Pathologic type, n (%)	
Adenocarcinoma	90 (90.91)
Squamous cell carcinoma	3 (3.03)
Mixed type	6 (6.06)
TNM stage, n (%)	
IIIc	3 (3.03)
IV	96 (96.97)
Sites of metastasis	
Brain	41 (41.41)
Liver	21 (21.21)
Bone	54 (54.55)
Primary *EGFR* mutation	
19del	50 (50.51)
L858R	42 (42.42)
others	7 (7.07)
Secondary T790M mutation	
Yes	28 (28.28)
No	37 (37.37)
Unknown	34 (34.34)
Previous EGFR-TKI treatment	
1^st^/2^nd^ generation TKI	44 (44.44)
1^st^/2^nd^ ➝3^rd^ generation TKI	48 (48.48)
3^rd^ generation TKI	7 (7.07)
Prior lines of therapy, n (%)	
≤2	43 (43.43)
>2	56 (56.57)
ICIs	
Anti-PD-1 antibody	93 (93.94)
Anti-PD-L1 antibody	6 (6.06)
Treatment	
Monotherapy	20 (20.20)
Combination therapy	
I+C	27 (27.27)
I+A	19 (19.19)
I+A+C	28 (28.28)
PD-1 inhibitor + CTLA-4 inhibitor	1 (1.01)
PD-1 inhibitor + EGFR-TKI	4 (4.04)

ECOG, Eastern Cooperative Oncology Group; EGFR, epidermal growth factor receptor; 19del, exon 19 deletion; ICI, immune checkpoint inhibitor; PD-1, programmed death 1; PD-L1, programmed cell death-ligand 1; I + C, Immunotherapy-chemotherapy combination treatment; I + A, Immunotherapy-antiangiogenic combination treatment; I + A + C, Immunotherapy-antiangiogenic-chemotherapy combination treatment; CTLA-4, cytotoxic T-cell lymphocyte-4; TKI, tyrosine kinase inhibitor.

### 3.2 Efficacy

At the time of data cutoff (February 11, 2022), the median duration of follow-up was 20.3 months (95% CI: 13.6-22.8) and 43 of 99 patients were still alive or lost to follow-up. A total of 81 patients had experienced disease progression. Thirty patients achieved PR as the best radiographic response, while 34 showed PD at the time of the first evaluation. No patients achieved CR. To compare with data in previous prospective studies, the patients receiving combination therapy with ECOG score 0-1 and no more than 2 lines of prior therapy were selected for analyze, and 26 patients met these criteria.

#### 3.2.1 ORR

Three patients had only non-target lesions. The ORR was 31.25% (30 of 96 patients; 95% CI: 22.18-41.52) in all patients with target lesions and 37.50% (9 of 24 patients; 95% CI: 18.80-59.41) in the selected patients. Further analyses were conducted to compare tumor response in different subgroups, and the results are presented in **Supplementary Table S1**. A significant difference in ORR was found in subgroups of different primary *EGFR* mutation types (*EGFR* 19del versus L858R versus others: 40.43% versus 16.67% versus 57.14%; *P*=0.012).

#### 3.2.2 DCR

The DCR was 65.66% (65 of 99 patients; 95% CI: 55.44-74.91) in all patients and 76.92% (95% CI: 56.35-91.03) in the selected 26 patients. The DCRs were significantly different according to age (<65 versus ≥65: 58.82% versus 80.65%; *P*=0.034) and treatment mode (monotherapy versus combination therapy: 40.00% versus 72.15%; *P*=0.007; **Supplementary Table S1**).

#### 3.2.3 PFS

**Figure 1:**
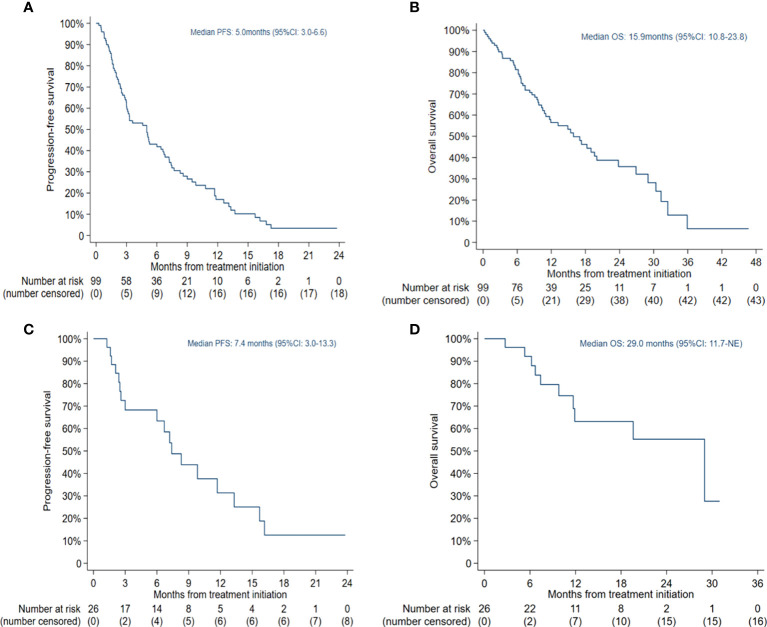
Kaplan-Meier estimates of progression-free survival (PFS) and overall survival (OS): **(A)** PFS in all patients; **(B)** OS in all patients; **(C)** PFS in patients receiving combination therapy with Eastern Cooperative Oncology Group (ECOG) score 0-1 and no more than 2 lines of prior therapy; **(D)** OS in patients receiving combination therapy with ECOG score 0-1 and no more than 2 lines of prior therapy.

**Figure 2:**
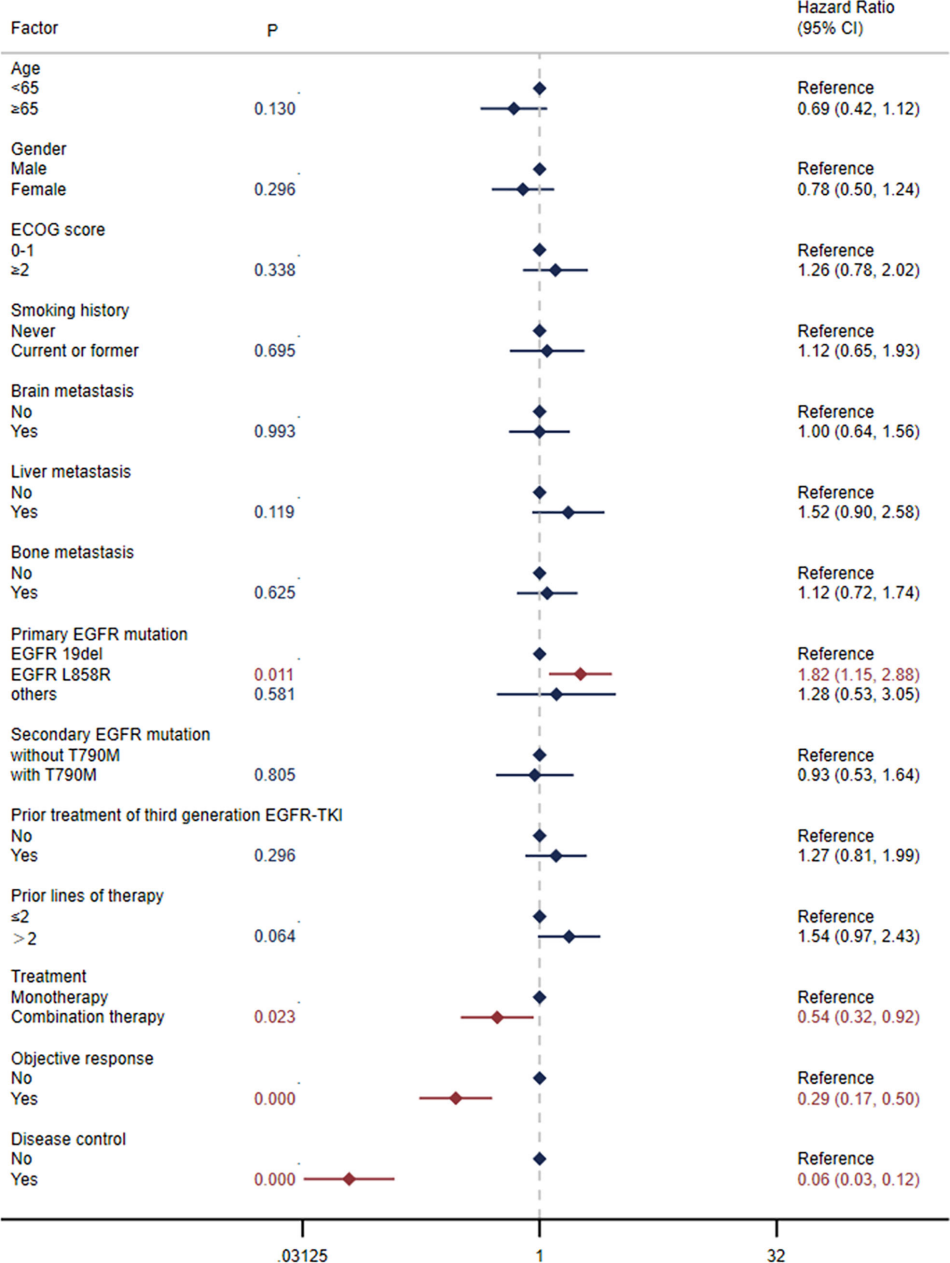
Univariate Cox regression analysis for progression-free survival (PFS).

The median PFS was 5.0 months (95% CI: 3.0-6.6) in all patients (**Figure 1A**) and 7.4 months (95% CI: 3.0-13.3) in the selected 26 patients (**Figure 1C**). PFS was found to be significantly correlated with primary *EGFR* mutation, treatment mode, objective response and disease control (**Figure 2**). In the multivariate model, primary *EGFR* mutation type and treatment mode demonstrated a significant association with PFS (**Figure 4**).

The median PFS was 2.5 months (95% CI: 1.9-5.0) in patients with *EGFR* L858R mutation versus 6.7 months (95% CI: 3.6-8.6) in patients with *EGFR* 19del mutation (HR=1.80, 95% CI: 1.14-2.86, log-rank *P*=0.011) (**Figure 5A**). In patients receiving combination therapy, the median PFS was 5.2 months (95% CI: 3.1-7.4), compared with 3.0 months (95% CI: 1.3-3.3) in patients receiving monotherapy (HR=0.54, 95% CI: 0.32-0.92, log-rank *P*=0.020; **Figure 6A**). To investigate the impact of the combination mode on PFS, the differences were further compared, and the results are shown in **Supplementary Figure S2A**. The I+A+C mode appeared to be superior. However, no significant differences were detected among the three combination groups.

#### 3.2.4 OS

**Figure 3:**
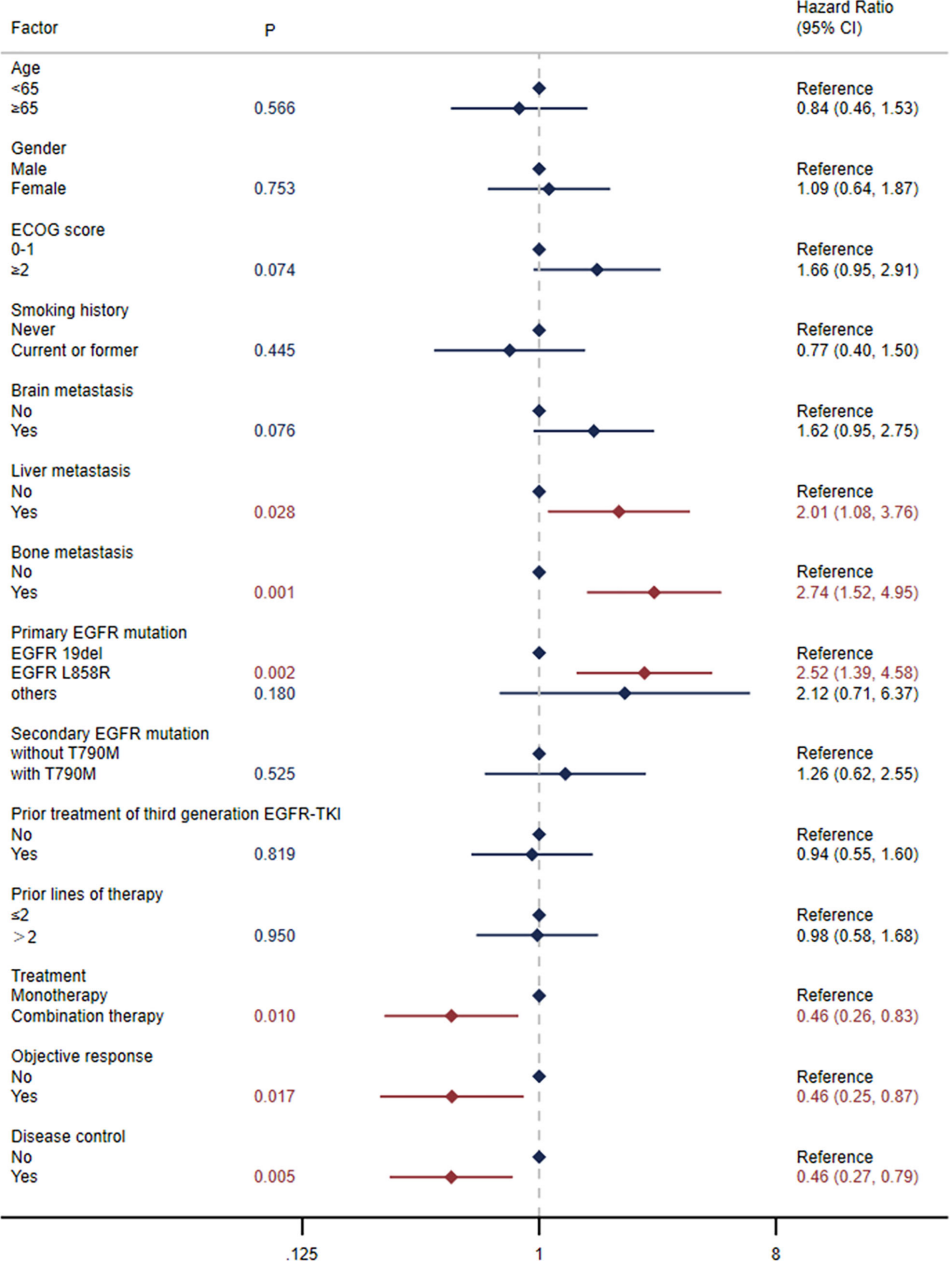
Univariate Cox regression analysis for overall survival (OS).

**Figure 4:**
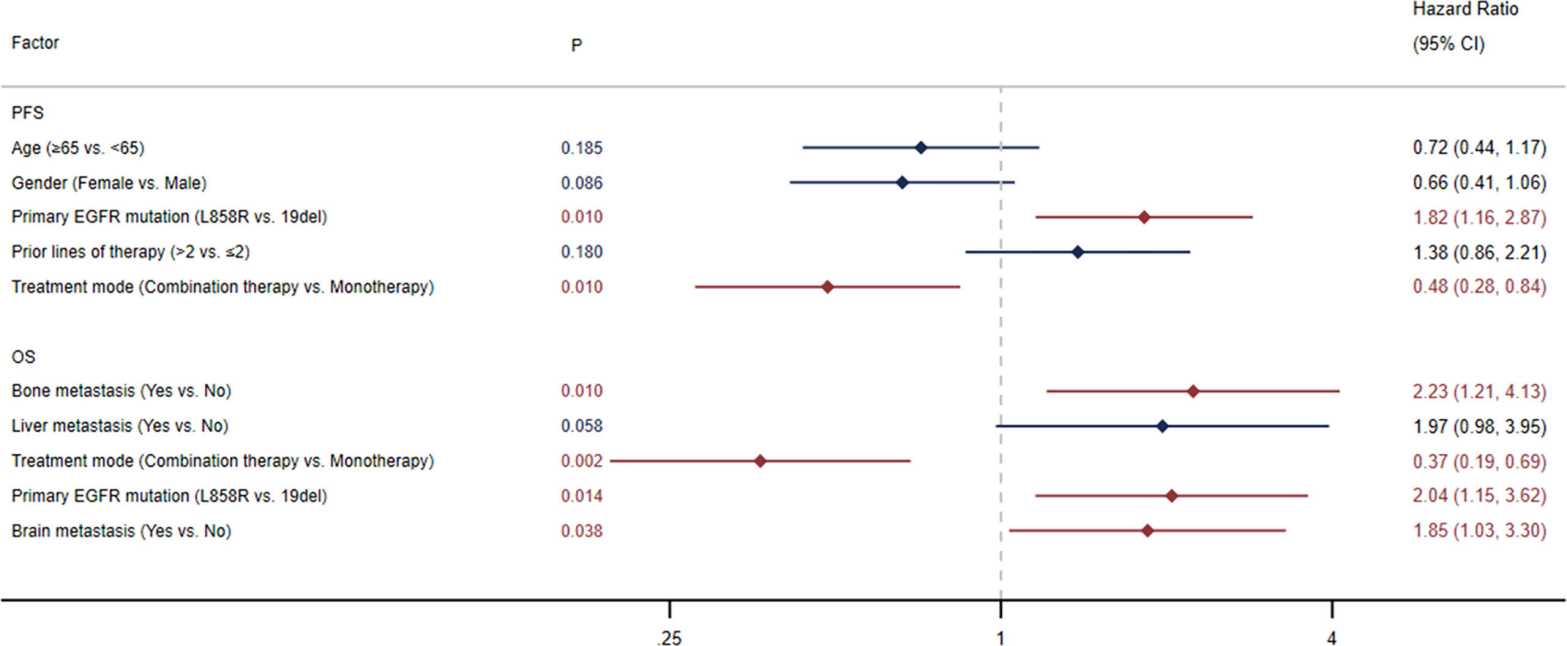
Multivariate Cox regression analysis for progression-free survival (PFS) and overall survival (OS).

The median OS was 15.9 months (95% CI: 10.8-23.8) in all patients (**Figure 1B**) and 29.0 months (95% CI: 11.7-NE) in the selected 26 patients (**Figure 1D**). OS was significantly correlated with primary *EGFR* mutation, treatment mode, status of liver metastasis, status of bone metastasis, objective response and disease control (**Figure 3**). In the multivariate model, primary *EGFR* mutation type, treatment mode, and status of bone metastasis and brain metastasis demonstrated a significant association with OS (**Figure 4**).

**Figure 5:**
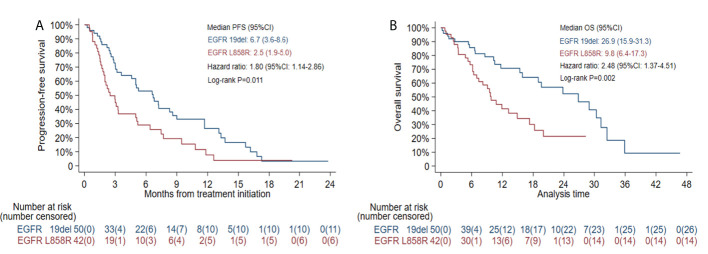
Kaplan-Meier estimates of progression-free survival (PFS) **(A)** and overall survival (OS) **(B)** in patients with EGFR exon 19 deletion (19del) or EGFR L858R mutation.

**Figure 6:**
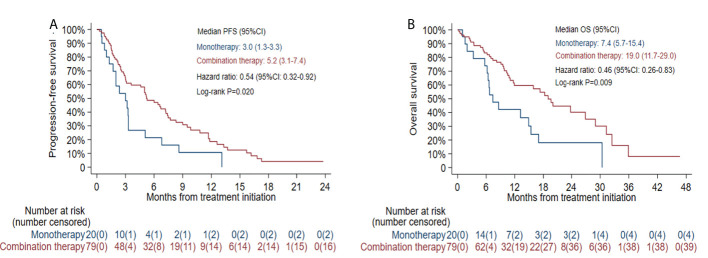
Kaplan-Meier estimates of progression-free survival (PFS) **(A)** and overall survival (OS) **(B)** according to different treatment modes.

The median OS was 9.8 months (95% CI: 6.4-17.3) in patients with an *EGFR* L858R mutation versus 26.9 months (95% CI: 15.9-31.3) in patients with an *EGFR* 19del mutation (HR=2.48, 95% CI: 1.37-4.51, log-rank *P*=0.002; **Figure 5B**). In patients receiving combination therapy, the median OS was 19.0 months (95% CI: 11.7-29.0) compared with 7.4 months (95% CI: 5.7-15.4) in patients receiving monotherapy (HR=0.46, 95% CI: 0.26-0.83, log-rank *P*=0.009; **Figure 6B**). The differences among different combination modes were further compared, and the results are shown in **Supplementary Figure S2B**. Similar to the result for PFS, the I+A+C mode appears to be superior, but no significant differences were detected among the three combination groups.

### 3.3 Adverse events

Thirty-two of 99 (32.32%) patients experienced Grade 3 and above TRAEs, including fatigue (n=15; 15.15%), neutropenia (n=13; 13.13%), thrombocytopenia (n=5; 5.05%), anemia (n=4; 4.04%), pneumonitis (n=3; 3.03%), vomiting (n=2; 2.02%), rash (n=2; 2.02%), increased alanine aminotransferase (n=1; 1.01%), and hypophysitis (n=1; 1.01%). The any grade irAEs were pneumonitis (n=8; 8.08%), hypothyroidism (n=5; 5.05%), rash (n=3; 3.03%), diarrhea (n=3; 3.03%), myositis (n=2; 2.02%), increased alanine aminotransferase (n=2; 2.02%), myocarditis (n=1; 1.01%), and hypophysitis (n=1; 1.01%). Grade 3 and above irAEs only occurred in 7 (7.07%) patients, including 3 pneumonitis (3.03%), 2 rash (2.02%), 1 increased alanine aminotransferase (1.01%) and 1 hypophysitis (1.01%). Two patients discontinued treatment due to irAEs. No treatment related death was observed.

## 4 Discussion

Few of real-world data focusing on ICI-based therapy in EGFR-TKI-resistant NSCLC have been reported. Our multicenter study with a relatively large sample size demonstrated a substantial benefit from ICI-based therapy in pretreated *EGFR*-mutated NSCLC in a real world setting. We found that the prognosis was significantly better in patients carrying the *EGFR* 19del mutation and receiving combination therapy than in those carrying the *EGFR* L858R mutation and receiving monotherapy.

*EGFR*-mutated NSCLC has been reported to be more likely to have an uninflamed tumor microenvironment (23), and PD-1 axis inhibition was once considered a blunt sword in this setting due to its poor effectiveness. However, on the basis of IMpower150, atezolizumab-bevacizumab-carboplatin-paclitaxel (ABCP) has been approved for *EGFR/ALK*-positive NSCLC after failure with TKIs in Europe. Immunotherapy has begun to show its edge in this setting and several clinical trials are ongoing. In IMpower 150, ABCP was reported to achieve a median OS of 27.8 months in sensitizing *EGFR*-mutated patients with prior TKI therapy (24). Recently, the phase-II trial of toripalimab plus chemotherapy as a second-line treatment in previously EGFR-TKI-treated patients with *EGFR*-mutant advanced NSCLC showed results with an overall ORR of 50.0% and DCR of 87.5%. The median PFS and OS were 7.0 and 23.5 months, respectively, in that study, which were superior to historical data of traditional chemotherapy (21). Tislelizumab combined with chemotherapy has been reported to achieve an overall ORR of 59.4% and DCR of 90.6% in a phase II trial (22). Compared with the results of previous studies on single-agent ICIs, these data from prospective clinical trials suggest that combination therapy may significantly improve prognosis despite a lack of direct comparison. In our study, we performed a comparison by using multicenter real-world data and our results support this hypothesis. However, whether differences exist among different combination modes needs to be explored. In addition, although no significant difference was detected according to different ECOG scores and prior lines of therapy, the median PFS and median OS in the selected patients receiving combination therapy with ECOG scores of 0-1 and no more than 2 lines of prior therapy were 7.4 months and 29.0 months, respectively, which is much better than the results in unselected patients. The longer PFS in selected patients suggests that ICI-based combination therapy might be more beneficial if it was applied as early as possible after EGFR-TKI resistance when patients had a better performance status.

Mounting evidence has supported the differences among *EGFR*-mutant subtypes. Although both 19del and L858R are classic *EGFR* mutations, they have different biological behaviors and responses to TKI treatment, as well as different resistance mechanisms to TKIs (25, 26). The differences in ICI efficacy between cases with *EGFR* 19del and those with *EGFR* L858R mutations are controversial. Our study indicated that patients with EGFR 19del could get more benefits than those with EGFR L858R. This difference might be explained by the potential differences in PD-L1 expression, tumor mutation burden (TMB) and the tumor microenvironment, such as CD8+ T-cell infiltration (18, 27, 28). Takada’s study retrospectively examined the relationship between PD-L1 expression and *EGFR* status in 441 surgically resected primary lung adenocarcinomas. They found that the prevalence of PD-L1 TPS 5-49% was higher among patients with an *EGFR* 19del than with an *EGFR* L858R mutation (28). On the other hand, Hastings’ study examined the molecular and clinical features of 171 *EGFR* mutant lung cancer cases treated with anti-PD-(L)1 alone or in combination with anti-CTLA-4 and found that outcomes were worse in patients with *EGFR* 19del but similar for *EGFR* L858R compared with *EGFR* wild-type NSCLC. They further assessed the relationship between TMB and specific *EGFR* alterations in a separate cohort of 383 *EGFR* mutant lung cancer cases irrespective of treatment exposure and found that *EGFR* 19del had a lower TMB than *EGFR* L858R lung tumors, which might account for the different response to ICIs (18). However, a study with 9649 Chinese primary NSCLC patients reported no significant differences in PD-L1 expression, TMB level or CD8+ T-cell infiltration between these two *EGFR* subtypes irrespective of treatment exposure (29). Notably, several studies have shown the impact of EGFR-TKI treatment on PD-L1 expression, TMB and the tumor immune microenvironment in *EGFR*-mutated NSCLC (30, 31). Isomoto et al. indicated that PD-L1 expression in tumor cells was significantly increased and TMB tended to be increased after resistance to EGFR-TKI treatment (30). Nishii et al. suggested that pretreatment with EGFR-TKI can induce CD8+ T-cell responses in *EGFR*-mutant NSCLC tumors, which was further pronounced by sequential dual blockade of PD-1 and vascular endothelial growth factor receptor 2 (31). All the above evidences demonstrate that the tumor immune microenvironment in *EGFR*-mutated NSCLC is dynamic. As a result, in order to explore the differences in ICI efficacy between EGFR subtypes, it is essential to detect potential predictive biomarkers at the same time point.

Some limitations exist in this study. First, this is a retrospective study, and some clinical data, such as PD-L1 expression are lacking. Although PD-L1 expression status is considered predictive for benefit from PD-1 or PD-L1 blockade therapy, it alone is insufficient in determining which patients should be offered PD-1 or PD-L1 blockade therapy (32). The PD-L1 expression during TKI treatment is dynamic just as above discussed. Further investigations are needed to examine this relationship and the potential mechanism. Second, various biases on the conditions of the patients exist, such as treatment mode, prior treatment lines and the performance status of the patients. The retrospective design and the sample size might lead to these biases. More large-scale studies are needed to establish the best combination mode. Third, there is not enough data to explore the relationship between comutations with EGFR and ICI efficacy in this kind of patient, as our previous study indicated comutation of EGFR and PI3K signaling had interaction effects on ICI treatment in nonsquamous NSCLC (33).

## 5 Conclusion

In conclusion, our study suggests that ICI-based combination therapy is a promising strategy in *EGFR*-mutated NSCLC after EGFR-TKI failure. The efficacy might be better in patients with *EGFR* 19del than in those with *EGFR* L858R.

## Data availability statement

The relevant data supporting the conclusions of this article will be available by contacting the corresponding authors upon reasonable request.

## Ethics statement

The studies involving human participants were reviewed and approved by the Ethics Committee of PLA General Hospital. Written informed consent for participation was not required for this study in accordance with the national legislation and the institutional requirements.

## Author contributions

Study design: XL, JM, and YH. Data collection: all authors. Data analysis: JH and DH. Manuscript writing: JH and YW. Manuscript editing and approval: all authors.

## Acknowledgments

Thanks to PLA General Hospital for supporting the study. Thanks all the patients and their families, and all the investigators involved in this study.

## Conflict of interest

The authors declare that the research was conducted in the absence of any commercial or financial relationships that could be construed as a potential conflict of interest.

## Publisher’s note

All claims expressed in this article are solely those of the authors and do not necessarily represent those of their affiliated organizations, or those of the publisher, the editors and the reviewers. Any product that may be evaluated in this article, or claim that may be made by its manufacturer, is not guaranteed or endorsed by the publisher.
