# Supplementary material for: The efficacy of immune checkpoint inhibitors in advanced EGFR-Mutated non-small cell lung cancer after resistance to EGFR-TKIs: Real-World evidence from a multicenter retrospective study
Source: Front Immunol. 2022 Sep 9;13:975246. doi: 10.3389/fimmu.2022.975246 (PMC9504865; doi:10.3389/fimmu.2022.975246)
Supplement: Supplementary file 1 [file DataSheet_1.docx]

Supplementary Material

# Supplementary Figures and Tables

## Supplementary Table

**Supplementary Table S1.** Correlation between tumor response and clinicopathological factors

|  | Objective response | | |  | Disease control | | | |
| --- | --- | --- | --- | --- | --- | --- | --- | --- |
|  | Yes | | No |  | Yes | | | No |
| Age |  | |  |  |  | | |  |
| <65 | 20 | | 46 |  | 40 | | | 28 |
| ≥65 | 10 | | 20 |  | 25 | | | 6 |
| X^2^/*P* value | 0.088/0.767 | |  |  | **4.497/0.034** | | |  |
| Gender |  | |  |  |  | | |  |
| Male | 13 | | 30 |  | 28 | | | 16 |
| Female | 17 | | 36 |  | 37 | | | 18 |
| X^2^/*P* value | 0.038/0.846 | |  |  | 0.143/0.705 | | |  |
| ECOG score |  | |  |  |  | | |  |
| 0-1 | 23 | | 44 |  | 47 | | | 23 |
| ≥2 | 7 | | 22 |  | 18 | | | 11 |
| X^2^/*P* value | 0.978/0.323 | |  |  | 0.234/0.628 | | |  |
| Smoking history |  | |  |  |  | | |  |
| Current or former | 8 | | 12 |  | 15 | | | 7 |
| Never | 22 | | 54 |  | 50 | | | 27 |
| X^2^/*P* value | 0.900/0.343 | |  |  | 0.080/0.777 | | |  |
| Brain metastasis |  | |  |  |  | | |  |
| Yes | 12 | | 27 |  | 25 | | | 16 |
| No | 18 | | 39 |  | 40 | | | 18 |
| X^2^/*P* value | 0.007/0.933 | |  |  | 0.680/0.410 | | |  |
| Liver metastasis |  | |  |  |  | | |  |
| Yes | 5 | | 14 |  | 14 | | | 7 |
| No | 25 | | 52 |  | 51 | | | 27 |
| X^2^/*P* value | 0.268/0.604 | |  |  | 0.012/0.913 | | |  |
| Bone metastasis |  | |  |  |  | | |  |
| Yes | 18 | | 33 |  | 36 | | | 18 |
| No | 12 | | 33 |  | 29 | | | 16 |
| X^2^/*P* value | 0.828/0.363 | |  |  | 0.054/0.817 | | |  |
| Primary *EGFR* mutation |  | |  |  |  | | |  |
| 19del | 19 | | 28 |  | 36 | | | 14 |
| L858R | 7 | | 35 |  | 24 | | | 18 |
| others | 4 | | 3 |  | 5 | | | 2 |
| X^2^/*P* value | **0.012^*^** | |  |  | 0.286^*^ | | |  |
| Secondary *EGFR* mutation |  | |  |  |  | | |  |
| with T790M | 7 | | 19 |  | 16 | | | 12 |
| without T790M | 12 | | 25 |  | 25 | | | 12 |
| X^2^/*P* value | 0.220/0.639 | |  |  | 0.744/0.388 | | |  |
| Prior treatment of 3^rd^ generation EGFR-TKI | | |  |  |  | | |  |
| Yes | 15 | 38 | |  | | 37 | 18 | |
| No | 15 | 28 | |  | | 28 | 16 | |
| X^2^/*P* value | 0.479/0.489 |  | |  | | 0.143/0.705 |  | |
| Prior lines of therapy |  | |  |  |  | | |  |
| ≤2 | 14 | | 27 |  | 29 | | | 14 |
| ＞2 | 16 | | 39 |  | 36 | | | 20 |
| X^2^/*P* value | 0.279/0.597 | |  |  | 0.108/0.743 | | |  |
| Treatment |  | |  |  |  | | |  |
| Monotherapy | 3 | | 17 |  | 8 | | | 12 |
| Combination therapy | 27 | | 49 |  | 57 | | | 22 |
| X^2^/*P* value | 0.064^*^ | |  |  | **7.317/0.007** | | |  |

*: Fisher’s exact test; ECOG: Eastern Cooperative Oncology Group; *EGFR*: epidermal growth factor receptor; 19del: exon 19 deletion.

## Supplementary Figures


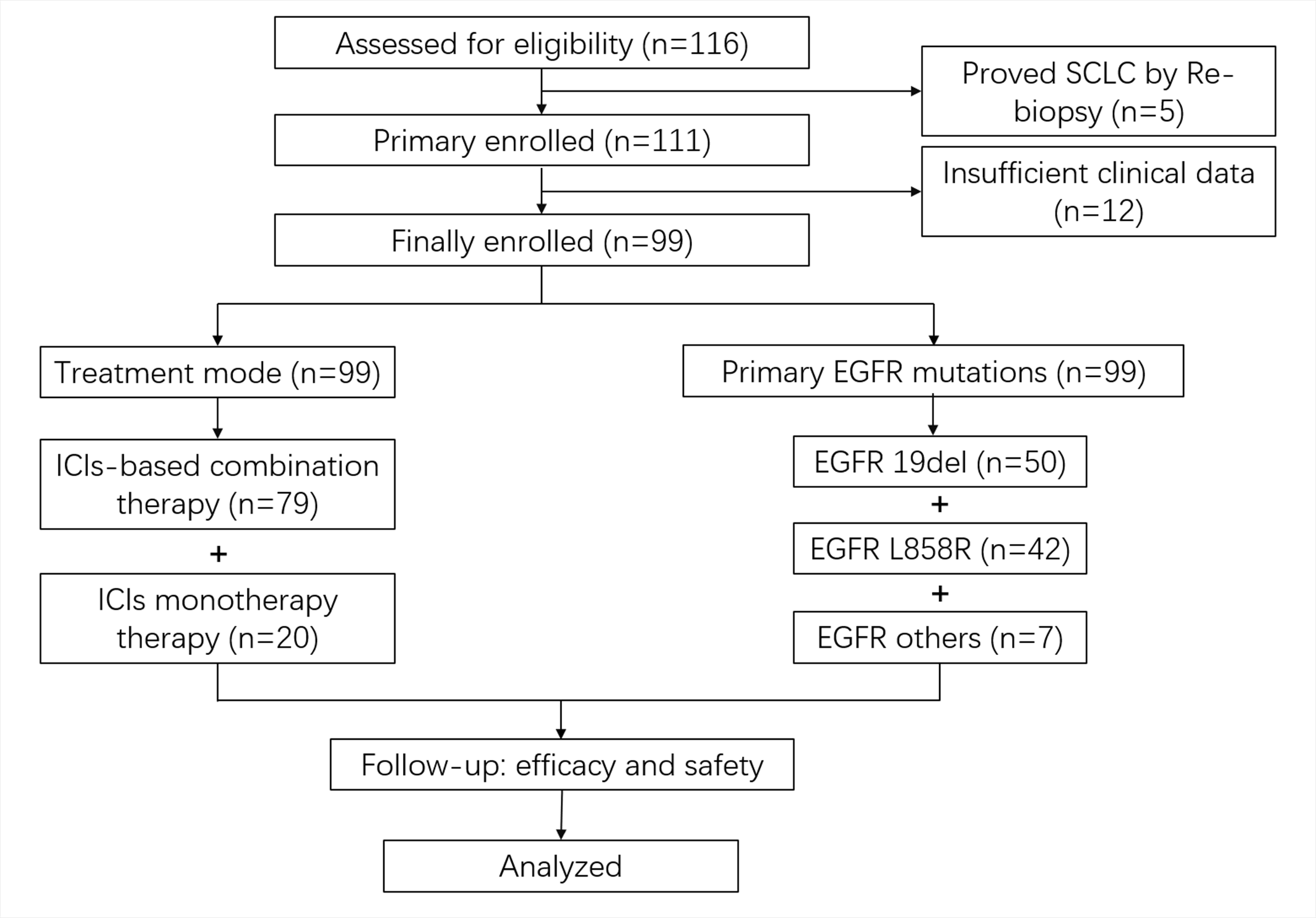


**Supplementary Figure S1.** Flow diagram.


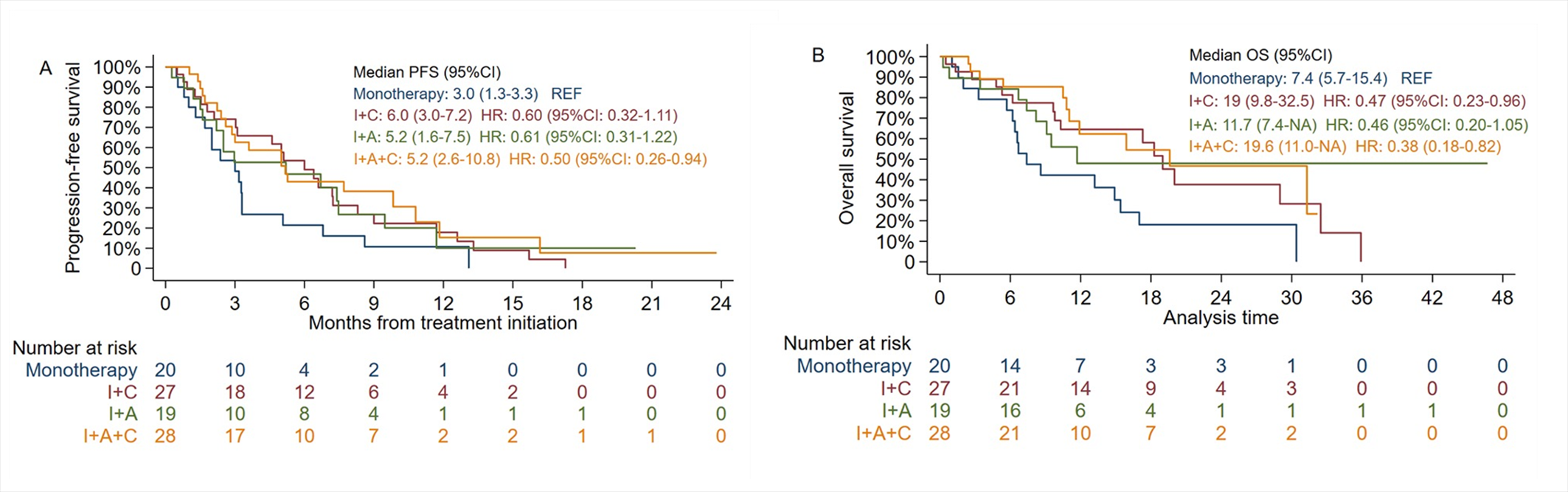


**Supplementary Figure S2.** Kaplan-Meier estimates of PFS (**A**) and OS (**B**) according to different combination mode.
